# Ternary crystal structure of human RORγ ligand-binding-domain, an inhibitor and corepressor peptide provides a new insight into corepressor interaction

**DOI:** 10.1038/s41598-018-35783-9

**Published:** 2018-11-26

**Authors:** Masato Noguchi, Akihiro Nomura, Satoki Doi, Keishi Yamaguchi, Kazuyuki Hirata, Makoto Shiozaki, Katsuya Maeda, Shintaro Hirashima, Masayuki Kotoku, Takayuki Yamaguchi, Yoshiaki Katsuda, Paul Crowe, Haiyan Tao, Scott Thacher, Tsuyoshi Adachi

**Affiliations:** 1 Pharmaceutical Frontier Research Laboratories, Central Pharmaceutical Research Institute, Japan Tobacco Inc., 1-13-2, Fukuura, Kanazawa-Ku, Yokohama, Kanagawa 236-0004 Japan; 2 Chemical Research Laboratories, Central Pharmaceutical Research Institute, Japan Tobacco Inc., 1-1, Murasaki-cho, Takatsuki, Osaka, 569-1125 Japan; 3 Biological Pharmacological Research Laboratories, Central Pharmaceutical Research Institute, Japan Tobacco Inc., 1-1, Murasaki-cho, Takatsuki, Osaka, 569-1125 Japan; 4 Orphagen Pharmaceuticals, 11558 Sorrento Valley Road, Suite 4, San Diego, California 92121 USA

## Abstract

Retinoic acid-related orphan receptor gamma (RORγ) plays pivotal roles in autoimmune diseases by controlling the lineage of interleukin 17 (IL-17)-producing CD4^+^ T cells (Th17 cells). Structure-based drug design has proven fruitful in the development of inhibitors targeting the ligand binding domain (LBD) of RORγ. Here, we present the crystal structure of a novel RORγ inhibitor co-complex, in the presence of a corepressor (CoR) peptide. This ternary complex with compound T reveals the structural basis for an inhibitory mechanism different from the previously reported inverse agonist. Compared to the inverse agonist, compound T induces about 2 Å shift of helix 5 (H5) backbone and side-chain conformational changes of Met365 on H5. These conformational changes correlate to reduced CoR peptide binding to RORγ-LBD in the presence of compound T, which suggests that the shift of H5 is responsible. This crystal structure analysis will provide useful information for the development of novel and efficacious drugs for autoimmune disorders.

## Introduction

T helper 17 (Th17) cells, which are a subset of CD4^+^ cells differentiated from naïve T cells, produces proinflammatory cytokines including interleukin 17 (IL-17). Th17 cells and the cytokine production play a pivotal role in autoimmune disease pathology in psoriasis, inflammatory bowel disease, rheumatoid arthritis and multiple sclerosis^1–6^. Anti-IL-17 monoclonal antibodies such as secukinumab and ixekizumab have been launched recently for the treatment of severe plaque psoriasis, psoriatic arthritis and ankylosing spondylitis^7,8^. Retinoic acid-related orphan receptor gamma (RORγ) is a key transcription factor and a master regulator of the differentiation of Th17 cells and the cytokine production. Orally available small-molecule inhibitors of RORγ have been created as a substitute medication for injectable biologics, which impose burdens to patients^9–11^.

**Figure 1:**
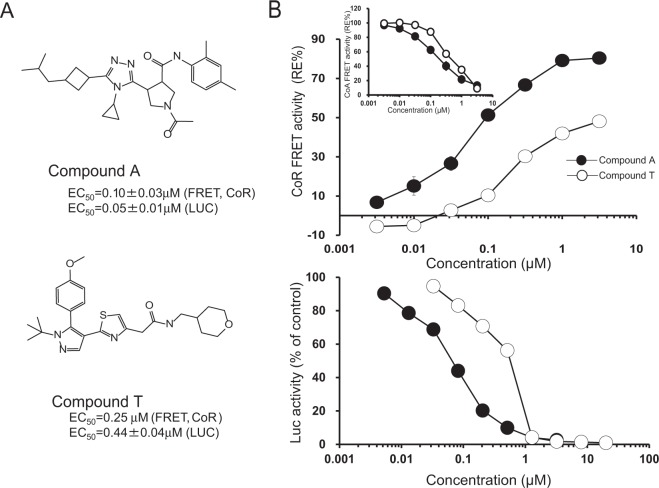
The relative efficacy (RE) of CoR peptide recruitment to the RORγ-LBD and LUC activity by inverse agonists. (**A**) Chemical structures of compound A and T. (**B**) Inverse agonist activities in FRET assays and LUC reporter assay. Inlet figure shows the CoA-releasing activities of inverse agonists. REs of CoR recruitment and CoA releasing are shown as percent activation relative to that of standard compound 1^9^. Background activity in the absence of a standard inverse agonist is defined as 0% response; and maximum activity induced by a standard inverse agonist at 100μM as 100% response. Dose-response curves are representative of independent results for each compound in LUC assay (Supplementary Fig. S1). A value of 100% is given to cells without inverse agonist in LUC assay. Data represent the means ± SE (n = 2–3), but not for data of compound T in FRET assay (n = 1).

Recently, we reported the first ternary structure of RORγ-LBD complexed with an inverse agonist, compound A (Fig. 1A), and a nuclear receptor corepressor (CoR) peptide, a silent mediator of retinoic acid receptor and thyroid receptor (SMRT) peptide (PDB code; 5X8X)^12^. Compound A was developed as a selective and orally efficacious RORγ inhibitor via structure-based drug design (SBDD) from a high-throughput screening (HTS) hit. It showed potent and efficacious activities in both a cell-based transcriptional assay and in a dual fluorescence resonance energy transfer (FRET)-based peptide interaction assay^13^. The FRET assay system used detects not only nuclear receptor coactivator (CoA) peptide releasing activity but also CoR peptide-recruiting activity^9,13^.

In the previous report, we showed that there were two types of inhibitors which induced C-terminus dislocations. Many RORγ inhibitors, such as ursolic acid (UA), hydrogen-bonded with His479 to restrict the movement of H11 in the C-terminus (PDB code; 5X8S)^14–21^. However, Compound A did not hydrogen-bond with His479 on H11, and it was therefore able to lead to larger dislocation of the C-terminus and give a ternary complex with a CoR peptide.

In this paper, we report a ternary structure in complex with a RORγ inhibitor from a different chemotype. This inhibitor, compound T, displays a unique structural mechanism for RORγ inhibition. As a result, compound T showed reduced potency in cell based transcriptional assay, whereas its potencies are comparable to compound A in dual FRET assay. Compound T is found to reduce efficacy in the FRET-based CoR recruitment assay. This ternary complex structure reveals the structural mechanism for the decreased CoR recruitment activity of RORγ in the presence of compound T when compared to compound A. The complex structure shows that the binding of compound T induces a movement of helix 5 (H5) that resulted in a decreased interaction interface between CoR peptide and the binding groove of RORγ-LBD. Significantly, these observations will allow us to design therapeutic inhibitors with advantageous biological and pharmacological activities.

## Results

### Activity measurement by FRET assay and LUC assays

Similar to compound A, compound T has dual activities in FRET assays, inducing the release of CoA peptide (EC_50_ = 0.5 µM) and the recruitment of CoR peptide (EC_50_ = 0.25 µM)^12^. The relative efficacy (RE) of compound T in CoR recruitment is about half that of compound A, but its potency is comparable to that of compound A (Fig. 1B). On the other hand, compound T shows similar potency and efficacy to compound A in FRET-based CoA-releasing assay. In luciferase (LUC) assay, EC_50_ of compound T is 0.44 µM, which is nine times lower activity than that of compound A (EC_50_ = 0.05 µM).

The CoR peptide-recruiting activity of rockogenin was not detected in dual FRET assay because of its low inhibitory activity, but the ternary complex was obtained in previous report^12^. The ternary complex crystal bound with rockogenin was used for a back-soaking replacement method because it has the lowest activity among compounds tested.

### The binding mode of compound T

Compound T, which is an antagonist of RORγ created by Yamamoto *et al*., inhibits the production of IL-17 in Th17 cells^22^. We expected that elucidating the complex structure with compound T would be instructive for designing synthetic inhibitors due to a different chemotype from compound A. Therefore, the ternary complex of RORγ-LBD with compound T and CoR peptide was determined at 2.6 Å resolution. As no crystals grew under the previous reservoir conditions for the RORγ-LBD, rockogenin crystals were soaked with compound T and CoR peptide. The data collection and the refinement statistics are summarized in Table 1.

**Table 1:** Crystal, data-collection and refinement statistics.

Complex type	Ternary
Ligand name	Compound T
**Data collection statistics**	
RORγ-LBD protein	mutant
Space group	*P*2_1_
a, b, c (Å)	77.89, 73.23, 100.13
α, β, γ (°)	90.00, 89.99, 90.00
Resolution range (Å)	77.89–2.55 (2.62–2.55)
Total reflections	133960
Unique reflections	35566
Completeness (%)	96.2 (79.8)
Redundancy	3.8 (3.8)
I/δ (I)	8.5 (1.4)
R_merge_ (%)	6.4 (56.2)
**Refinement statistics**	
Resolution range (Å)	2.55–77.93
No. of reflections	34576
R_cryst_ (R_free_)	21.50 (23.91)
**No**. **of atoms**	
Protein	7866
Ligand	132
Water	91
**B-factors**	
Protein	59.27
Ligand	51.21
Water	61.08
**r**.**m**.**s**. **deviations**	
Bond length (Å)	0.016
Bond angles (°)	1.785

Values in parenthesis are for the highest resolution shell.

Rmerge = ∑|*I*_h_ − (*I*)_h_|/∑*I*_h_, where (*I*)_h_ is average intensity over symmetry equivalents.

R-factor = ∑|*F*_obs_ − *F*_calc_|/∑*F*_obs_. The free R-factor is calculated from 5% of the reflections that are omitted from the refinement.

**Figure 2:**
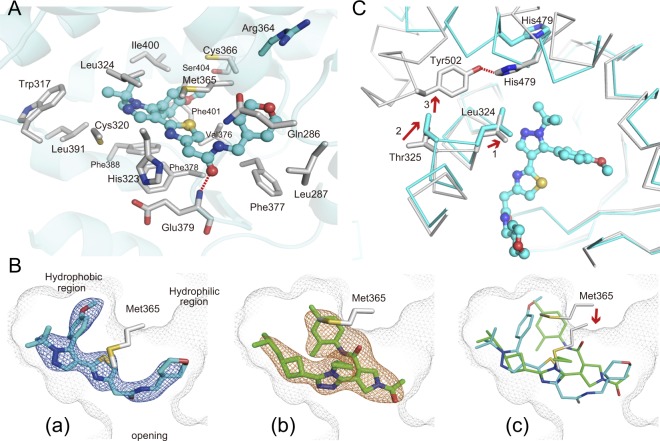
The binding mode and inhibitory mechanism of compound T. (**A**) Detailed interaction of compound T in the ligand-binding pocket. Compound T forms one hydrogen-bond with a main-chain nitrogen atom of Glu379. Side chains of the RORγ-LBD with less than 4 Å distance towards compound T are shown as *gray* sticks. Side chain of Arg364 in the hydrophilic side pocket is shown in *cyan*. Compound T is shown in ball-and-stick representation depicted in *cyan*. Nitrogen, oxygen and sulfur atoms are depicted in *blue*, *red* and *camel*, respectively. Hydrogen-bonds are indicated in *red* dotted line. (**B**) Superimposition of compound T to compound A in the ligand-binding pocket. Backbone of Met365 shifts towards compound T (*red* arrow). Compound A and compound T are shown as *green* and *cyan* sticks, respectively. The electron densities for compound A and compound T in the *mFo-DFc* omit maps are shown as *orange* mesh for compound A and *blue* mesh for compound T (contoured at 2.5σ). Pale *gray* mesh shows the outline of the pocket. (**C**) Possible inhibitory action of compound T emerged by superimposing the inhibitor conformation (*cyan*) to the active conformation (*gray*) (PDB code; 5X8W)^12^. Compound T induces the dislocation of H11′ and H12 by initiating an interaction with Leu324. Leu324 shifts towards the compound (*red* arrow1) and the concerted movement of Thr325 (*red* arrow2) leads to a clash with Tyr502 in the active conformation (*red* arrow 3), resulting in disruption of the hydrogen-bond (*red* dotted line) between His479 and Tyr502 (*gray*) to form the inhibitor binding conformation. The C-terminal region from H11′ to H12 involving Tyr502 is not visible in the inhibitor binding conformation.

In the ternary complex of RORγ-LBD bound with the compound T, the ligand omit map obtained from the refined model showed an extra electron density modeled as compound T (Fig. 2B). Extensive interactions of compound T with RORγ-LBD include a direct hydrogen-bonding and van der Waals (VdW) contacts. O atom in the amide group of compound T accepts a hydrogen-bonding from backbone N atom of Glu379 (Fig. 2A). The sixteen amino acid residues exhibiting non-hydrogen-bonding atoms that are closer than 4 Å towards compound T are as follows: Gln286, Leu287, Trp317 (H4), Cys320 (H4), His323 (H4), Leu324 (H4), Met365 (H5), Cys366 (H5), Val376, Phe377, Phe378, Phe388 (H6), Leu391 (H6), Ile400 (H7), Phe401 (H7) and Ser404 (H7). Compound T has the same number of VdW contacts as compound A in the binding pocket.

**Figure 3:**
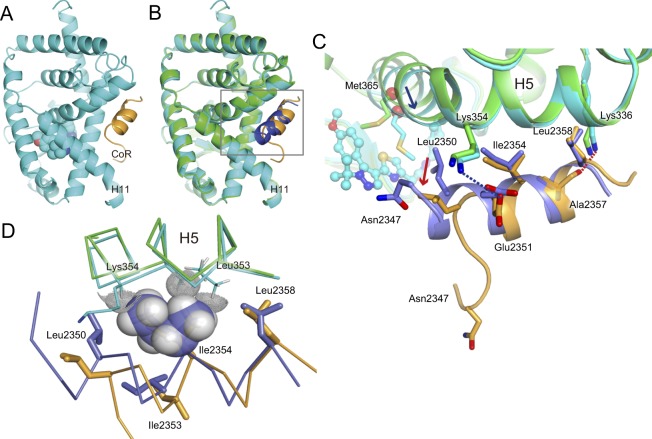
The ternary complex of the RORγ-LBD containing compound T and SMRT22 peptide. (**A**) Overall ternary complex of the RORγ-LBD (*cyan*) containing compound T and CoR peptide (*camel*). The C-terminus region involving H11′ and H12 is disordered. Compound T in the cannonical orthosteric ligand-binding pocket is depicted in *cyan* spheres. (**B**) Superimposition of the ternary complex RORγ-LBD/compound A/CoR peptide (RORγ in *green*, CoR in *blue*) to that of RORγ-LBD/compound T/CoR peptide (RORγ in *cyan*, CoR in *camel*). For clarity, compounds are not shown. (**C**) Close-up view of square in panel B. Unwound N-terminus region of CoR peptide is observed in the complex structure with compound T. Cα positions at residue Met365 in H5 are shown as *red* balls. H5 in the ternary complex bound with compound T moves toward compound (*blue* arrow), and N-terminus region of CoR is disordered. Ile2354 and Leu2358 form hydrophobic interactions with the RORγ-LBD, but Leu2350 presumably loses contact (*red* arrow). Ionic interaction between Glu2351 and Lys354 is depicted in *blue* dotted line. Possible electrostatic interaction between the nearest Ala2357 backbone carbonyl and Lys336 side chain is shown in *red* dotted line. (**D**) Steric hindrance of shifted H5 to CoR peptide. Backbone of H5 moves towards CoR peptide. Leu353 and Lys354 clash with Ile2354. Ile2354 is depicted as semi-transparent solid sphere. Hydrogen atoms of Leu353 side chain and Lys354 backbone are depicted as dot-spheres. Color scheme follows that of C.

An amide group of compound T orientates towards the opening of the ligand binding pocket with *tert*-butyl-pyrazole group lying in the hydrophobic residues lining the pocket (Figs 2A, 2B). Tetrahydropyran group of the compound locates the hydrophilic side pocket consisted by Gln286 and Arg364, in the vicinity of the opening. In this binding mode, thiazole ring of the compound interacts with Met365, which locates between tetrahidropyran and methoxy-benzen group and interacts with both functional groups. This interaction induces not only the large shift of Met365 on H5, but also approximately 2 Å movement of H5 backbone toward the compound when compared with compound A complex (Figs 2B, 3C). The large movement of H5, which is not observed in the complex structure with compound A and rockogenin previously reported, occurred in the back-soaking crystal containing rockogenin and CoR peptide.

The double mutant (dm) Lys469Ala/Arg473Ala of RORγ-LBD enabled us to obtain the ternary structure bound with compound A and the corepressor SMRT22 (22 mer) peptide as described in a previous report^12^. The obtained ternary structure can reflect in a rational antagonist design on unique mechanism in terms of recruiting CoR peptide. The dm RORγ-LBD also gives the complex structure with higher frequency than wild-type, despite different chemotypes^12^. In case of compound T, no crystal grew in ternary crystal structure of the dm RORγ-LBD bound with compound and CoR peptide.

### Compound T induces larger dislocation in C-terminus like compound A

Two types of C-terminus dislocations were observed when inhibitors bind to RORγ-LBD (PDB codes; 5X8X, 5X8S)^12^. Compound A induced larger dislocation in C-terminus than UA because there was no interaction between compound A and His479, which restricted the movement of H11. Therefore, CoR peptide could bind to the hydrophobic surface emerged by the larger dislocation in C-terminus. As for the lager displacement mechanism, compound T will display the same mechanism as compound A. The small movement of Leu324 toward compound T in the ternary complex is also observed by superimposition to the apo (non-liganded) conformation. The interaction between compound T and Leu324 will be a trigger of displacement of the C-terminal. By way of the direct hydrophobic interactions to Leu324, a concerted shift of Thr325 causes consequently steric-interference to Tyr502, which will disrupt the hydrogen-bonding in the active conformation in the same manner as with compound A (Fig. 2C)^12^. This is the mode of inhibitory action of compound T, which has indirect effect on hydrogen-bonding disruption between His479 and Tyr502. In this conformation, compound T does not form the hydrogen-bonding with His479 on H11. Superimposition to the apo conformation also indicates that any shifts of amino-acid residues observed by the shift of Met365 involving movement of H5 backbone do not cause steric hindrance with His479 or Tyr502.

### Compound T indirectly reduces CoR peptide interaction towards RORγ-LBD

The overall ternary structure of RORγ-LBD in complex with compound T is seemingly the same as that in complex with compound A (Fig. 3A,B, Supplementary Fig. S2). Similar to compound A^12^, the C-terminus region of H11 is observed in a displaced position from the canonical active H11 position, and a structure with the corresponding residues downstream from Val493 (H11′) to H12 shows no traces of these residues in the electron density maps. The invisible structure will imply dislocation of C-terminus induced by compound T and it will lead to release of CoA peptide and recruitment of CoR peptide. When precisely compared to the ternary structure bound with compound A, binding mode of SMRT 22 peptide to RORγ-LBD seems to be different. The observed N-terminus region upstream from Glu2351 is unwound and Leu2350 backbone shifts approximately 2 Å outwards from RORγ-LBD. Asn2347 in the N-terminal of SMRT peptide moves approximately 15 Å from the SMRT 22 in complex with compound A. SMRT 22 peptide binds to the hydrophobic groove of RORγ-LBD emerged by the dislocation in C-terminus. Ile2354 and Leu2358 interact hydrophobically with RORγ-LBD (Fig. 3C), but Leu2350 does not. Leu353 and Lys354 backbones of H5 move 0.6 Å towards CoR peptide. Leu353 side chain and Lys354 backbone clash with original site of Ile2354 (Fig. 3D). Glu2351 side chain slightly shifts, but interacts ionically with Lys354 (H5) side chain. A backbone carbonyl group of Ala2357 retains the same site and keeps a weak electrostatic interaction from Lys336 (H3) side chain. Compound T is located more than 10 Å away from CoR peptide, thus it can have no direct interaction with CoR peptide.

## Discussion

Compound T shows a dual-activity that releases CoA peptide and recruits CoR peptide like compound A. The EC_50_ values on FRET activities are almost the same as for compound A, but compound T shows less RE than compound A on FRET-based CoR-recruiting assay. Moreover, compound T demonstrates much less activity than compound A in LUC assay compared to that in the FRET assay. To investigate the mode of action of compound T, a ternary structure of hRORγ-LBD complexed with compound T and SMRT 22 peptide was determined and compared with the previously reported ternary complex with compound A. Overall structure comparison between compound T and compound A displays H5 movement and displacement at one extremity of CoR peptide. The ternary complex bound with compound T explains the reduced CoR binding from a perspective of structural biology. Compound T induces the dislocated N-terminus of CoR peptide in a binding mode that will induce less interaction with hRORγ-LBD than that with compound A (Fig. 3C). The conformational change of the CoR peptide will be the cause of the lower efficacy on the FRET assay (Fig. 1B).

Compound T induces a large conformational change in ligand binding pocket. Met365 on H5 shifts towards compound T and its backbone moves towards compound T. We presume that the Leu2350 shift and large movement of Asn2347 are caused by the movement of H5 in comparison to compound A, because compound T does not directly interact with CoR peptide. As for PPARα, the Leu2350Ala mutation decreased binding activity by 50%, but the Asn2347Ala mutation did not affect the interaction of CoR^23^. The CoR clearly shows less interaction than that of the ternary complex with compound A by losing the contact with Leu2350.

We assume that the conformational change of the CoR peptide will be induced by the large movement of H5, which in turn appears to cause the lower efficacy in the FRET assay. Since no movements of H5 are observed with both compound A and rockogenin as well as in the apo conformation, the volumes of pockets in compound A and rockogenin are similar to apo conformation in size (~700Å^3^). However, the volume of pocket in compound T is smaller than that in apo conformation by approximately 30%^24,25^. This is clearly due to the movement of H5, the effect of which reaches out to the binding surface with CoR peptide (Fig. 3C,D). Activity enhancement has been achieved on the derivatives from compound A, which does not induce the H5 movement (preparing papers for publication). All these compounds achieved the same efficacy at higher concentration. Novel compounds with higher FRET efficacy and higher LUC activity may be synthesized from compound T if the conformational disadvantage can be overcome by structure-activity relation (SAR) analysis. Actually, it has been reported recently that some related compounds can be improved without the movement of H5^26^.

Orally administered compound A effectively suppressed IL-17 release in a dose-dependent manner in inflammatory model mice^9^. Compound A displayed the highest activities on both dual FRET assay and LUC assay among our tested compounds. In the series of compound A, the activities on dual FRET assay were parallel to the activities on LUC assay. Compound T not only showed half the efficacy of compound A on the FRET-based CoR recruitment assay, but was also nine-times lower than compound A on LUC activity (EC_50_ of compound T = 0.44 μM, EC_50_ of compound A = 0.05 μM). Compound T shows similar potency and efficacy to compound A in FRET-based CoA-releasing assay (Fig. 1B). The reduced LUC activity compared to the FRET-based CoR-recruiting activity suggests that compound T may exert stronger effect on CoR-binding ability in the cells. Similar to compound A, compound T has no direct contact with the CoR peptide. These results suggest that compound T indirectly reduces the complete interaction of the CoR to the binding groove of RORγ-LBD in the cells.

Many RORγ inhibitors are reported as inverse agonists without showing CoR peptide-recruiting activities or crystal structures of ternary complex. It is not clear whether these inhibitors should be classified as antagonist or inverse agonist. The endogenous ligands of RORγ can bind to RORγ-LBD and function as agonists in biological assays such as LUC assays^27^. Ligand-mediated CoR interactions are an important aspect for regulating transcriptional activity of nuclear receptors^23,28,29^. However, no biochemical studies using two-hybrid or pull-down analyses have ever been reported to investigate whether RORγ can interact with SMRT or other CoRs. Therefore, we expect that comparison of compounds A and T could lead to further biochemical or functional understanding of CoR behavior in cells, and these could also be compared to UA, which shows no CoR-recruiting activity in the FRET assay. When CoRs recruited to RORγ on the promoter repress the transcription of target genes, HDAC3 may be involved in histone deacetylation on chromatin condensation. It is interesting to speculate whether inhibitor-mediated recruitment of CoRs to RORγ induces transcriptional repression through chromatin repression.

Finally, this report considers CoR peptide binding mode on inhibitory activity through ternary complexes of human RORγ-LBD. We clarified the important points for activities of inverse agonists by knowing the actions of inverse agonists on the C-terminus dislocation and on the consequent CoR peptide binding. We believe that our novel structural knowledge will lead to safe and effective new therapeutic agents for RORγ, which plays a crucial role in Th17-drived autoimmune disorders.

## Methods

The compound T, generated as working example 39 by another company, was selected from some patents and the potency of a drug having a different chemotype from ours was investigated^22^. Detailed methods on plasmid construction, sample preparation, crystallization, structure determination, and FRET and LUC assays are provided in previous publications^12^. Briefly, the site-directed mutants of the RORγ-LBD (260–518aa) cloned into pET28 plasmid were produced in *Escherichia coli*, and purified by Ni-NTA affinity column and gel-filtration. The purified proteins were crystallized in complex with rockogenin and CoR peptide (ternary complex structures). For ternary complex of compound T, crystals of rockogenin complex obtained by sitting-drop vapor-diffusion method were soaked with 10 mM inhibitor and 1 mM CoR peptide (PDB code; 5X8Q)^12^. The complex structures were determined by molecular replacement method using the RORγ-LBD apo structures as a search model. Diffraction data of the RORγ-LBD were collected at 21IDF (APS), Chicago, USA. Figures were generated using PyMOL^30^.

For TR-FRET assays, the glutathione-S-transferase (GST)-tagged RORγ-LBD produced in Sf9 cells and peptide solution were incubated with compounds, and the 520/490 TR-FRET ratio was measured. Human RORγ LUC assays were carried out in accordance with the method of Madoux *et al*.^31^ with some modifications and described previously^9^. EC_50_ was calculated by curve-fitting in GraphPad Prism. Detailed descriptions of synthesis for the compounds are described elsewhere^9,22^.

## Electronic supplementary material

Supplementary Information

## Data Availability

The atomic coordinates and structure factors in this paper have been deposited in the Protein Data Bank (PDB code: 6A22).
