# Supplementary material for: Ternary crystal structure of human RORγ ligand-binding-domain, an inhibitor and corepressor peptide provides a new insight into corepressor interaction
Source: Sci Rep. 2018 Nov 26;8:17374. doi: 10.1038/s41598-018-35783-9 (PMC6255837; doi:10.1038/s41598-018-35783-9)
Supplement: Supplementary file 1 — Supplementary Information [file 41598_2018_35783_MOESM1_ESM.pdf]

Supplementary information

Ternary crystal structure of human RORγ ligand-binding-domain, an inhibitor and corepressor peptide provides a new insight into corepressor interaction

Authors:

Masato Noguchi[1*], Akihiro Nomura[1], Satoki Doi[1], Keishi Yamaguchi[1], Kazuyuki Hirata[2], Makoto Shiozaki[2], Katsuya Maeda[2], Shintaro Hirashima[2], Masayuki Kotoku[2], Takayuki Yamaguchi[3], Yoshiaki Katsuda[3], Paul Crowe[4], Haiyan Tao[4], Scott Thacher[4] and Tsuyoshi Adachi[1*]

Affiliations:

[1] Pharmaceutical Frontier Research Laboratories, Central Pharmaceutical Research Institute, Japan Tobacco Inc., 1-13-2, Fukuura, Kanazawa-Ku, Yokohama Kanagawa, 236-0004, Japan

[2] Chemical Research Laboratories, Central Pharmaceutical Research Institute, Japan Tobacco Inc., 1-1, Murasaki-cho, Takatsuki Osaka, 569-1125, Japan

[3] Biological Pharmacological Research Laboratories, Central Pharmaceutical Research Institute, Japan Tobacco Inc., 1-1, Murasaki-cho, Takatsuki Osaka, 569-1125, Japan

[4] Orphagen Pharmaceuticals, 11558 Sorrento Valley Road, Suite 4, San Diego, California, 92121, United States

*Correspondence should be addressed to M.N. (masato.noguchi@jt.com) or T.A. (tsuyoshi.adachi@jt.com)

Masato Noguchi and Akihiro Nomura contributed equally to this work.

Supplementary Figures

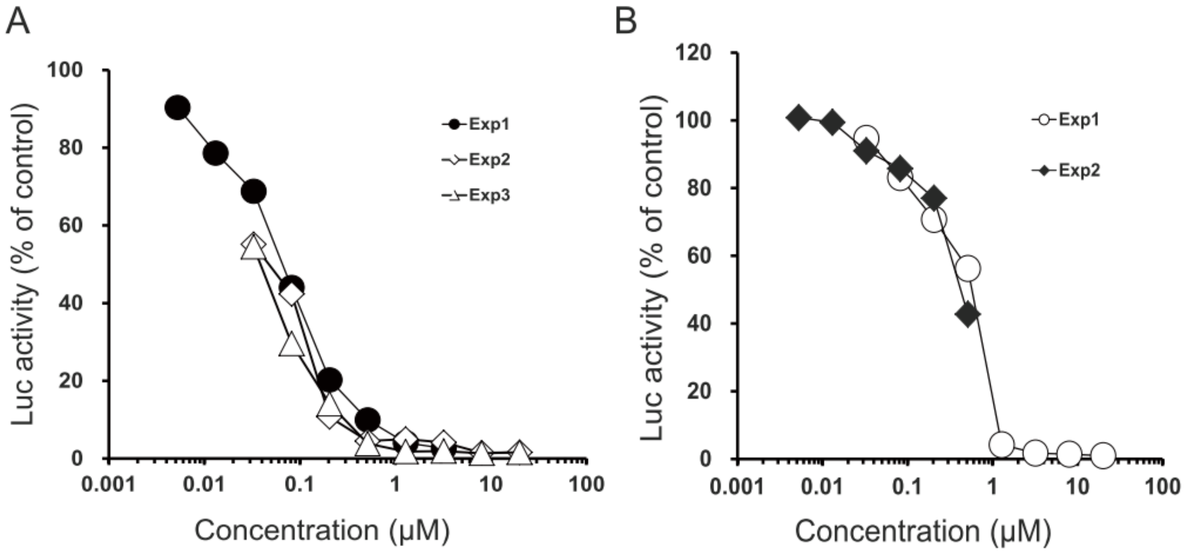

Figure S1. Inverse agonist activities in LUC reporter assay.

(A) Three independent experiments of inhibitory activity of compound A are overlaid.

(B) Two independent experiments of inhibitory activity of compound T are overlaid.

High-reproducibility was observed in LUC assay system.

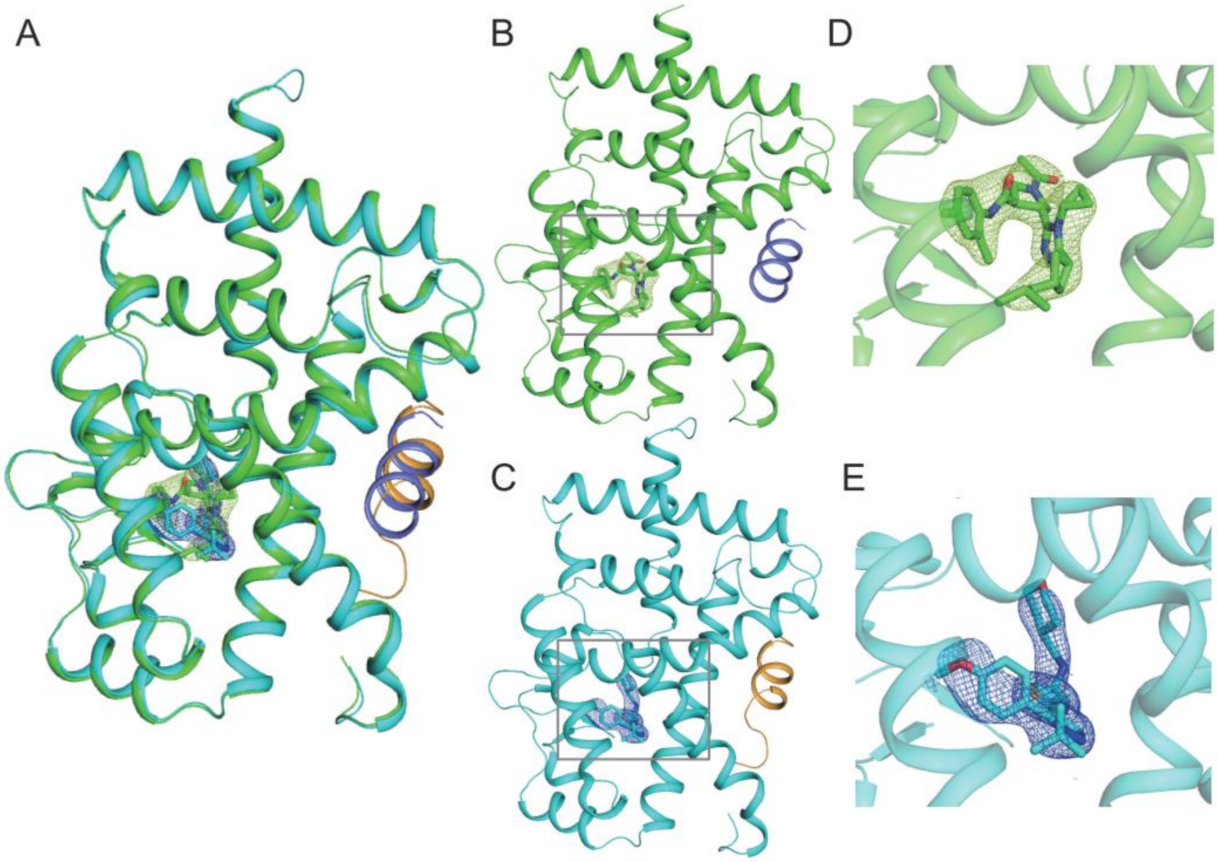

Figure S2. The ternary complexes of the RORγ-LBD containing compounds and SMRT22 corepressor (CoR) peptide.

(A) Superimposition of the ternary complex RORγ-LBD/compound A/CoR peptide (RORγ in *green*, CoR in *blue*) to that of RORγ-LBD/compound T/CoR peptide (RORγ in *cyan*, CoR in *camel*). Electron density maps (*mFo-DFc*) of compounds are shown at 1σ.

(B)(C) Ternary complexes of RORγ-LBD/compound A/CoR peptide and RORγ-LBD /compound T/CoR peptide, respectively.

(D)(E) Close up views of squares in panel B and C, respectively.
